# A growth-based screening strategy for engineering the catalytic activity of an oxygen-sensitive formate dehydrogenase

**DOI:** 10.1128/aem.01472-24

**Published:** 2024-08-28

**Authors:** Feilong Li, Silvan Scheller, Michael Lienemann

**Affiliations:** 1 Department of Bioproducts and Biosystems, Aalto University, Espoo, Finland; 2 VTT Technical Research Centre of Finland Ltd., Espoo, Finland; University of Milano-Bicocca, Milan, Italy

**Keywords:** enzyme engineering, growth-based screening, metalloenzyme, formate dehydrogenase, oxygen sensitivity, formate hydrogenlyase

## Abstract

Enzyme engineering is a powerful tool for improving or altering the properties of biocatalysts for industrial, research, and therapeutic applications. Fast and accurate screening of variant libraries is often the bottleneck of enzyme engineering and may be overcome by growth-based screening strategies with simple processes to enable high throughput. The currently available growth-based screening strategies have been widely employed for enzymes but not yet for catalytically potent and oxygen-sensitive metalloenzymes. Here, we present a screening system that couples the activity of an oxygen-sensitive formate dehydrogenase to the growth of *Escherichia coli*. This system relies on the complementation of the *E. coli* formate hydrogenlyase (FHL) complex by Mo-dependent formate dehydrogenase H (*Ec*FDH-H). Using an *Ec*FDH-H-deficient strain, we demonstrate that growth inhibition by acidic glucose fermentation products can be alleviated by FHL complementation. This allows the identification of catalytically active *Ec*FDH-H variants at a readily measurable cell density readout, reduced handling efforts, and a low risk of oxygen contamination. Furthermore, a good correlation between cell density and formate oxidation activity was established using *Ec*FDH-H variants with variable catalytic activities. As proof of concept, the growth assay was employed to screen a library of 1,032 *Ec*FDH-H variants and reduced the library size to 96 clones. During the subsequent colorimetric screening of these clones, the variant A12G exhibiting an 82.4% enhanced formate oxidation rate was identified. Since many metal-dependent formate dehydrogenases and hydrogenases form functional complexes resembling *E. coli* FHL, the demonstrated growth-based screening strategy may be adapted to components of such electron-transferring complexes.

**IMPORTANCE:**

Oxygen-sensitive metalloenzymes are highly potent catalysts that allow the reduction of chemically inert substrates such as CO_2_ and N_2_ at ambient pressure and temperature and have, therefore, been considered for the sustainable production of biofuels and commodity chemicals such as ammonia, formic acid, and glycine. A proven method to optimize natural enzymes for such applications is enzyme engineering using high-throughput variant library screening. However, most screening methods are incompatible with the oxygen sensitivity of these metalloenzymes and thereby limit their relevance for the development of biosynthetic production processes. A microtiter plate-based assay was developed for the screening of metal-dependent formate dehydrogenase that links the activity of the tested enzyme variant to the growth of the anaerobically grown host cell. The presented work extends the application range of growth-based screening to metalloenzymes and is thereby expected to advance their adoption to biosynthesis applications.

## INTRODUCTION

Enzyme engineering has been instrumental in transforming wild-type enzymes into potent catalysts for the synthesis of commodity chemicals from highly concentrated non-natural substrates at elevated temperatures and in the presence of organic solvents (1). The methods employed for such enzyme customizations are either of a rational nature or introduce random mutations in the gene encoding the protein of interest. When the latter approach is combined with a selection step and iterated over several rounds, the process mimics natural selection and is, therefore, called directed evolution (2). The advantage of this method is that it requires little knowledge of the enzyme structure and its catalytic mechanism but depends on high-throughput screening (HTS) methods for identifying enzyme variants with improved properties (3). The development of these screening techniques has been the primary challenge in most enzyme engineering campaigns and is limited by the low amount of assay principles that are compatible with automated sample processing in the HTS format, i.e., being chiefly fluorometric and colorimetric (3, 4). A wider application of HTS methods for enzyme variant screening is hindered by the requirement for elaborate sample handling (e.g., induction of gene expression, cell fragmentation, and analysis of enzymatic activity) and expensive microfluidic devices or robotic platforms (5).

Growth-based screening is a powerful strategy for screening large variant libraries with easily measurable readout, high throughput, and minimal equipment dependence (2, 6, 7). The design of growth-based screening approaches is challenging because it requires a strong link between the fitness of a host organism and the mutations introduced into a gene of interest (6, 7). A widely employed growth-based screening method is based on auxotrophic host organisms where the introduction of catalytically active enzyme variants complements the disrupted metabolic functions and can be readily identified by measuring the growth of their host cells (6). This approach has proven useful for improving natural amino acid production [e.g., chorismate mutases and amino acid racemases (8, 9)], switching cofactor dependency [e.g., metal-free formate dehydrogenases (10) and NAD(P)H oxidases (11)], and increasing the activity for the synthesis of non-natural chiral amines [e.g., hydrolases (12, 13), amine transaminases, ammonia lyase, and monoamine oxidase (7)]. Noteworthy, other growth-based screening methods employ, e.g., the production of variants of detoxifying enzymes or the inclusion of fitness-enhancing reporter genes that are transcribed following the binding of a complex between a gene regulator and a molecule synthesized by the enzyme of interest (e.g., β-lactam hydrolases and benzaldehyde dehydrogenases) (14–16). An underrepresented group of biocatalysts in enzyme engineering campaigns are oxygen-sensitive enzymes, such as metal-dependent formate dehydrogenases, hydrogenases, and nitrogenases. These enzymes are of high interest to industrial applications owing to their bound soft, electron-rich metal ions allowing the transfer of electrons to chemically inert electron acceptor molecules such as CO_2_ and N_2_ (17). Relevant fields of active research include the hydrogen and formate economy as well as green nitrogen fixation and production of chemical feedstocks (18–20). The metal centers employed by these redox-active enzymes are easily inactivated by molecular oxygen. Therefore, screening of the coordinating enzymes can only be performed in formats that allow maintenance of strictly anoxic assay conditions and, at the same time, are robust and scalable. Growth-based screening methods fully comply with these requirements and are, therefore, ideally suited for the engineering of oxygen-sensitive metalloenzymes. So far, no screening strategy has been reported that links cell growth to the catalytic function of such enzymes and, thereby, prevents their engineering in a HTS format.

**Fig 1:**
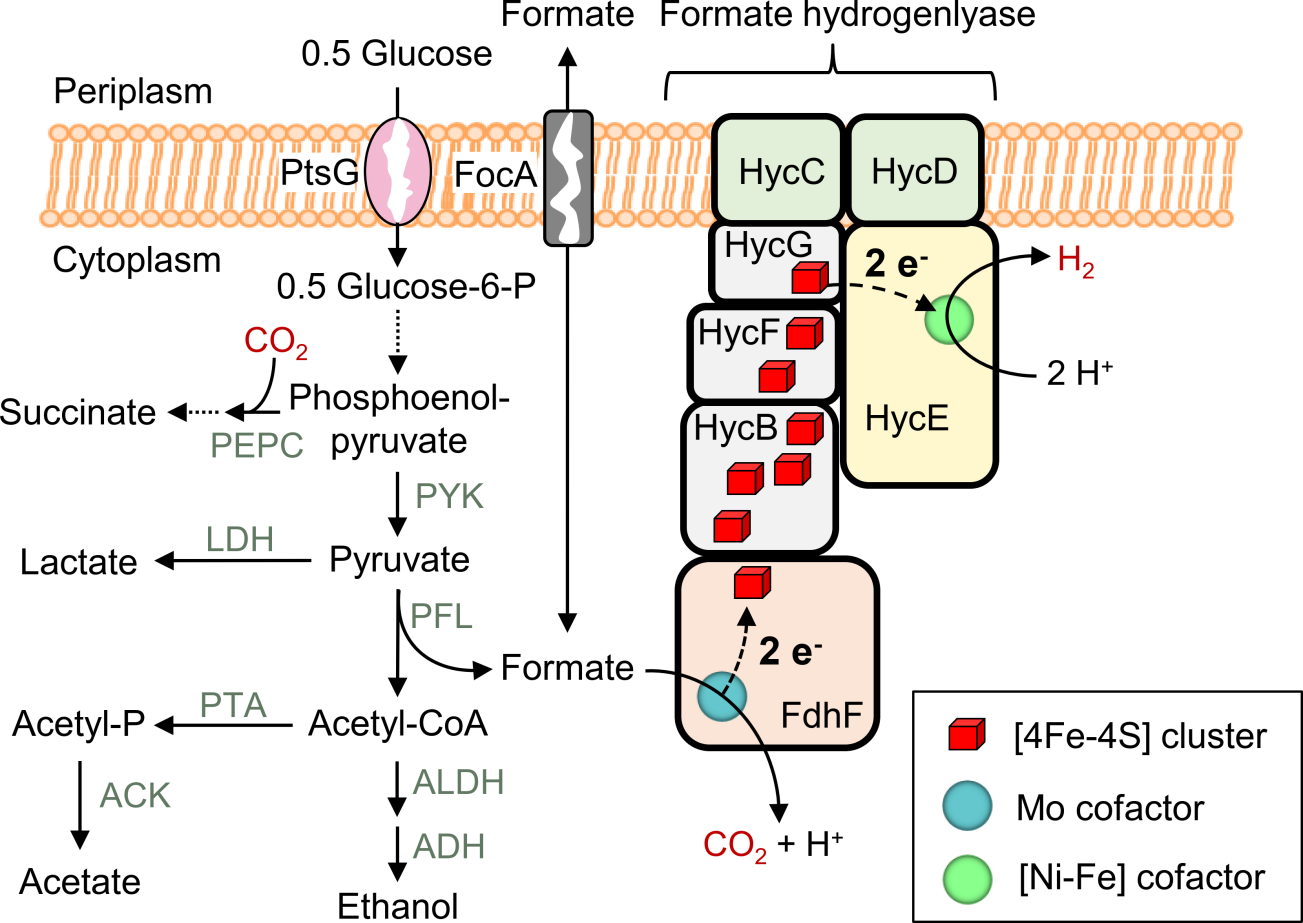
Mixed-acid fermentation yielding succinate, ethanol, acetate, formate, and lactate. The shown reactions are catalyzed by phosphoenolpyruvate carboxylase (PEPC), pyruvate kinase (PYK), lactate dehydrogenase (LDH), pyruvate formate lyase (PFL), phosphotransacetylase (PTA), acetate kinase (ACK), acetate dehydrogenase (ADH), and acetaldehyde dehydrogenase (ALDH). Formate is disproportionated into CO_2_ and H^+^ by the formate hydrogenlyase complex. Here, two electrons are released at the formate dehydrogenase H subunit (FdhF, referred to as *Ec*FDH-H in the main text), passed on to the hydrogenase 3 (HycE) subunit via an array of eight iron–sulfur clusters, and ultimately transferred onto two protons to yield H_2_. The membrane-bound glucose-specific phosphotransferase system (PtsG) and formate channel (FocA) facilitate the passage of glucose and formate, respectively, across the cytoplasmic membrane.

Here, we present a growth-based screening method for the identification of oxygen-sensitive variants of *Escherichia coli* formate dehydrogenase H (*Ec*FDH-H) with increased catalytic activity. The screening system relies on the complementation of the membrane-bound formate hydrogenlyase (FHL) complex employing the *Ec*FDH-H enzyme as an electron input module to couple intracellular formate oxidation to proton reduction (Fig. 1). The production of functional *Ec*FDH-H variants alleviates growth inhibition by formate and proton accumulation during fermentative growth and enabled the screening of an enzyme variant library with cell density as a simple readout. This screening method significantly reduced the library size and was combined with a colorimetric assay in order to identify variants with improved catalytic activity.

## RESULTS AND DISCUSSION

### Correlating FDH-H activity to fermentative metabolism of *E. coli*

#### Effect of *Ec*FDH-H removal on cell viability

**Fig 2:**
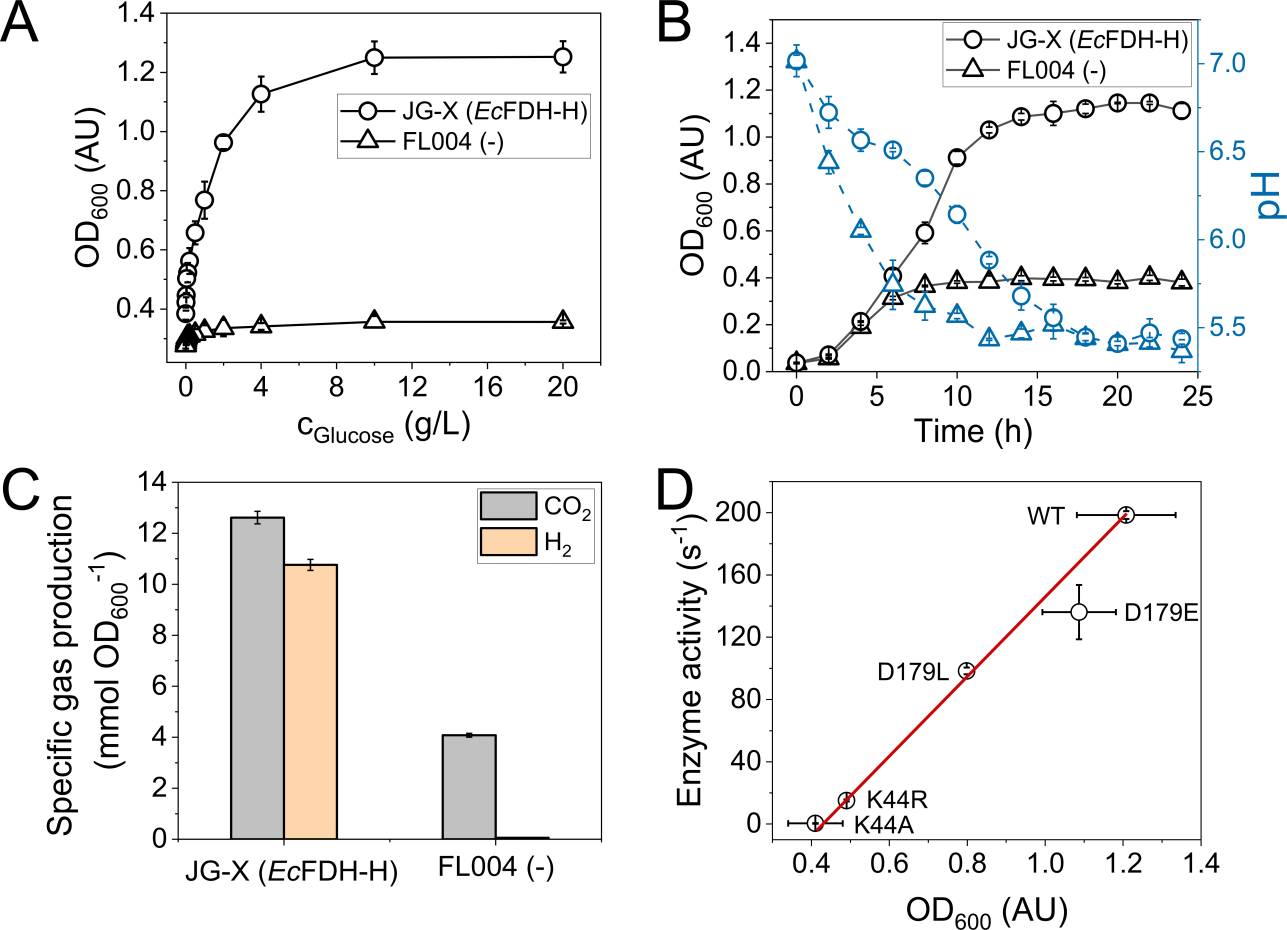
Effects of FHL complementation by *Ec*FDH-H on anaerobic growth of *E. coli* with glucose measured as (**A**) final cell density at varying glucose concentrations, (**B**) time-dependent change of cell density and culture medium pH at *c*_Glucose_ = 4 g/L, and (**C**) gas production at *c*_Glucose_ = 4 g/L. (**D**) Correlation of final host cell density reached after 24-h anaerobic fermentation of glucose and *in vitro* formate oxidation activity of *Ec*FDH-H. The activities of *Ec*FDH-H enzymes (wild type, D179E, and D179L: 1.5 µg; K44R and K44A: 7.5 µg) were determined spectroscopically at λ = 555 nm as BV^2+^ reduction using a phosphate-buffered reaction mix (pH 7.5) containing the substrates formate and BV^2+^ at a concentration of 10 and 2 mM, respectively. Error bars represent the standard deviation of three independent experiments.

The presented screening strategy relies on *E. coli* growth during glucose fermentation as a measure of the activity of a formate dehydrogenase bound to HycB as an electron-donating component of the FHL complex (Fig. 1). In order to establish the importance of the functional FHL complex for fermentative growth, the *Ec*FDH-H-producing and -deficient *E. coli* strains JG-X and FL004, respectively (Table 1), were cultivated under anaerobic atmosphere at varying glucose concentrations. The strain JG-X was included as a reference, owing to its Δ*fdhF* and Δ*iscR* genotype. The *fdhF* deletion eliminates the *Ec*FDH-H background activity of the *E. coli* MC4100 wild-type strain while the production of the other two formate dehydrogenases *Ec*FDH-O and *Ec*FDH-N is maintained at natural levels. The *iscR* gene encodes an iron–sulfur cluster regulator that represses the transcription of the *iscRSUA* operon (21) and was deleted in the *Ec*FDH-H production strains in order to increase the cofactor occupancy and production level of Fe-S cluster-containing proteins as reported for the production of 4-hydroxy-3-methylbut-2-enyl diphosphate reductase in *E. coli* (22, 23). As evident from Fig. 2A, strain JG-X reached a final cell density (OD_600_) of 1.25 ± 0.05 while strain FL004 did not exceed an OD_600_ value of 0.357 ± 0.006 revealing a strong growth retardation effect of the *Ec*FDH-H deletion. The retarded fermentative growth of strain FL004 may be explained by the compromised function of its FHL complex, i.e., remediation of the cytosol acidification and induction of cellular stress resulting from the accumulation of weak organic acids during mixed acid fermentation (Fig. 1) (24, 25). Notably, *Ec*FDH-H deletion did not result in growth retardation under aerobic conditions (Fig. S1) because these catabolic intermediates are not formed if oxygen is used as the exclusive terminal electron acceptor. Formate is among these fermentation products and can enter the cell via membrane-embedded transporters such as FocA (26). It also induces the synthesis of the membrane-bound FHL complex that catalyzes formate disproportionation into CO_2_ and H_2_ (27, 28). Possible formate cytotoxicity was investigated by the exposure of the *E. coli* strains FL004 and JG-X to varying concentrations of formate. Here, strain FL004 was found to be more sensitive to formate when compared to the *Ec*FDH-H-producing control JG-X (Fig. S2A). This result is consistent with the faster growth of the parental strain JG-X during glucose fermentation (Fig. 2A) and primary formate degradation by the FHL complex (Fig. 1). The extent to which *Ec*FDH-H contributes to deacidification during fermentative growth was assessed by monitoring the pH of anaerobic *E. coli* cultures and evolution of hydrogen gas. The culture broth pH of the *Ec*FDH-H-deficient strain FL004 decreased from 7.0 to below 6.1 within 4 h while the strain JG-X maintained the culture medium pH above 6.1 and grew exponentially for up to 10 h (Fig. 2B). These results are in line with a previous study by Beyer et al. who reported retarded fermentative growth and similar pH profiles for a *selC* deletion strain of *E. coli* (29). In contrast, a more recent study by Gevorgyan et al. found *E. coli fdhF* deletion to decrease the final cell density during glucose fermentation by merely ~10% (30). We attribute this to the presence of 100-mM phosphate buffer in the growth media, which maintained the external media pH at ≥6.4 during their examined 72-h cultivation period. Therefore, the accelerated growth medium acidification of the *Ec*FDH-H-deficient strain observed in the current study can be explained by the interruption of electron flow through the FHL complex to the HycE subunit catalyzing proton reduction to molecular hydrogen.

**TABLE 1:** Derivatives of *Escherichia coli* strain JG333 (MC4100 *ΔfdhF, ΔiscR*) used for the production of *Ec*FDH-H and its variants^*b*^

Strain	Plasmid-encoded FDH	Plasmids	VTT-CC ID
JG-X^*a*^	*Ec*FDH-H	pSU21-*selC*, pTrc99a-*fdhF*	E-223614
FL003	–^*c*^	pSU21-*selC*	E-233617
FL004	–^*c*^	pSU21-*selC,* pTrc99a	E-233618
FL005	*Ec*FDH-H-A12G	pSU21-*selC*, pFL003	E-233619
FL006	*Ec*FDH-H-D179E	pSU21-*selC*, pFL004	E-233620
FL007	*Ec*FDH-H-D179L	pSU21-*selC*, pFL005	E-233621
FL008	*Ec*FDH-H-K44R	pSU21-*selC*, pFL006	E-233622
FL009	*Ec*FDH-H-K44A	pSU21-*selC*, pFL007	E-233623

^*a*^ Source: Prof. Shelley Minteer (Utah State University, Utah) (31).

^*b*^ VTT Culture Collection strain identifiers (VTT-CC IDs) are indicated.

^*c*^ –, neither the native nor the mutated *fdhF* gene is contained in the harbored plasmid(s).

#### Effect of EcFDH-H removal on intracellular proton reduction

FHL-dependent hydrogen production was investigated by the collection and analysis of the gas that was produced by *E. coli* strains JG-X and FL004 during 24 h of fermentative growth. The strain JG-X produced CO_2_ and H_2_ at a molar ratio that was slightly different from 1:1 (Fig. 2C; Table S1) and indicated that the produced gas did not exclusively originate from the coupled formate oxidation and proton reduction at the FHL complex. A similar excess of released CO_2_ has been reported earlier for glucose fermentation in a minimal growth medium by the *E. coli* strain MG1655 (32). The excess of CO_2_ may result from varying activities of CO_2_-releasing and consuming enzymes, such as the periplasmic FDHs *Ec*FDH-O and *Ec*FDH-N (28) as well as phosphoenolpyruvate carboxylase (PEPC) (33), respectively (Fig. 1). In the present study, cultivations were performed in a complex growth medium that contains amino acids, and their enzymatic decarboxylation may contribute to the observed CO_2_ liberation. The *E. coli* strain JG-X produced 1.9 mmol OD_600_^−1^ CO_2_ more than H_2_, and the *Ec*FDH-H deletion practically abolished H_2_ production and increased the CO_2_ excess to 4.0 mmol OD_600_^−1^. This finding may be linked to FHL inactivation as it may lead to periplasmic formate accumulation and thereby boost formate oxidation by *Ec*FDH-O and *Ec*FDH-N. As opposed to FHL, these enzymes are not electrically linked to a hydrogenase, but electrons are instead transferred to the cytochrome *bd* oxidase I and nitrate reductase via the quinone pool of the cytoplasmic membrane giving rise to water and nitrite as electron transfer products, respectively, instead of H_2_ (34, 35). While an active PEPC may decrease the amount of released CO_2_ (Fig. 1), an apparent lack of succinate in the product spectrum of glucose-fermenting *E. coli* indicates that CO_2_ consumption via this metabolic route is negligible (32). *Ec*FDH-H deletion reduced the volume of produced gas from 761 ± 8 mL (JG-X) to 34.7 ± 8.3 mL (FL004) and diminished the hydrogen fraction from 10.8 ± 0.2 mmol OD_600_^−1^ to 57.5 ± 0.6 µmol OD_600_^−1^ (Fig. 2C). This corresponds to a reduction of the detected amount of hydrogen down to 0.53% in the case of the *Ec*FDH-H-deficient strain and is suspected to be not a product of microbial metabolism but rather a result from the exposure of the cell culture to a hydrogen-containing atmosphere in the anaerobic cabinet that could not be completely removed by purging with nitrogen gas. Importantly, the complete abolition of hydrogen production upon FHL complex disruption is in line with earlier studies according to which the majority of hydrogen produced during fermentative *E. coli* growth is produced by the HycE hydrogenase subunit of the FHL complex that depends on *Ec*FDH-H as the source of electrons (36–43). In addition, the presented results suggest that recombinantly produced *Ec*FDH-H can effectively complement an incomplete FHL complex and that *Ec*FDH-H extension with a C-terminal His_6_-tag does not disturb electron transfer within the FHL complex. The latter finding is consistent with the FHL complex structure in which the C-terminus of the *Ec*FDH-H subunit and the HycB–*Ec*FDH-H interface are separated 23 Å from each other [Fig. S3 (44)] allowing for correct complex assembly.

#### Correlation of cell density and Ec*FDH*-H activity

Inspired by the distinctive growth characteristics of *E. coli* strains JG-X and FL004, we hypothesized that the activity of an *Ec*FDH-H variant may be estimated using the final cell density reached by *Ec*FDH-H variant-producing *E. coli* cells during 24 h of glucose fermentation. To test this idea, the residues K44 and D179 were selected as substitution targets based on their respective degree of conservation among metal-dependent FDHs. Specifically, K44 is highly conserved and its substitution was expected to result in a large activity loss, while the aspartate at position 179 is less conserved and its substitution may be expected to result in minor enzyme activity changes. A correlation analysis was performed with *Ec*FDH-H variants -K44A, -K44R, -D179L, and -D179E exhibiting formate oxidation activities below the wild-type level of 199 s^−1^ and between 0.4 s^−1^ and 136 s^−1^. A good correlation between enzyme activity and final cell density was apparent for all tested *Ec*FDH-H variants with the exception of D179E, which slightly deviated from the overall trend toward a decreased enzyme activity or higher cell density (Fig. 2D). The reasons for this are not obvious but may include an increased expression level of the gene encoding *Ec*FDH-H-D179E gene compared to the wild-type *fdhF* gene and an altered electron transfer from the variant to BV^2+^ that may decrease the apparent enzyme activity in the cell-free assay. Similar deviations from a linear relationship between cell growth and *in vitro* enzyme activity have been reported for an NADPH-dependent d-lactate dehydrogenase (45).

### Screening of an *Ec*FDH-H variant library by FHL complex complementation

#### Screening assay development and validation

**Fig 3:**
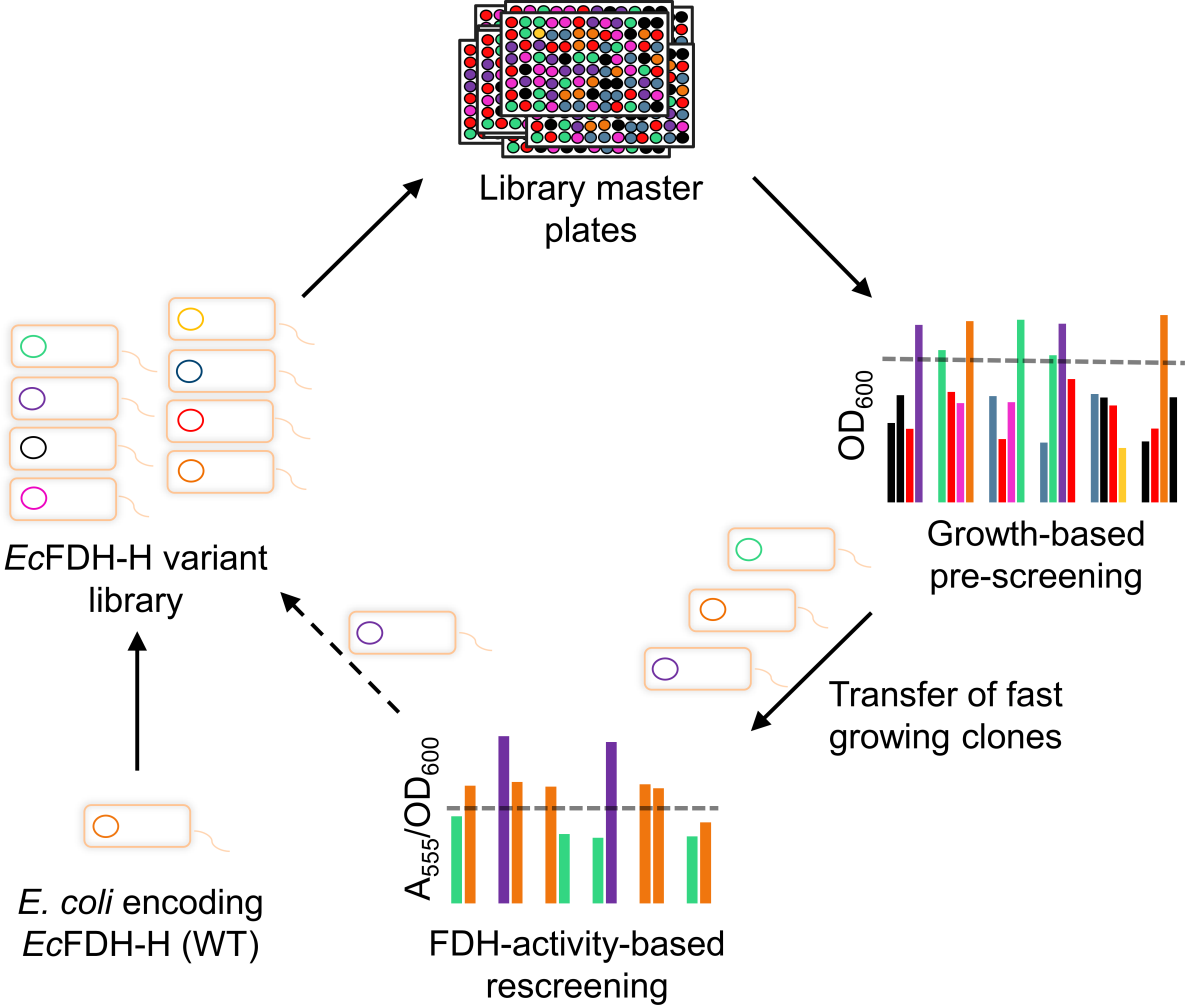
Schematic of a two-step anaerobic screening platform for the engineering of *Ec*FDH-H. Firstly, a clone library of *E. coli* strains encoding different variants of *Ec*FDH-H is prepared aerobically by focused or random mutagenesis and distributed into wells of library master plates. The screening process is performed under anaerobic conditions in a 96-well plate format and initiated by pre-screening the entire clone library using the final optical density (OD_600_) as a selection criterion. Clones exceeding an OD_600_ threshold (dashed line) calculated from the *Ec*FDH-H-producing strain JG-X are subjected to FDH activity-based rescreening, and the clones exhibiting improved enzyme activity can be selected for an optional additional round of gene mutagenesis and two-step screening.

**Fig 4:**
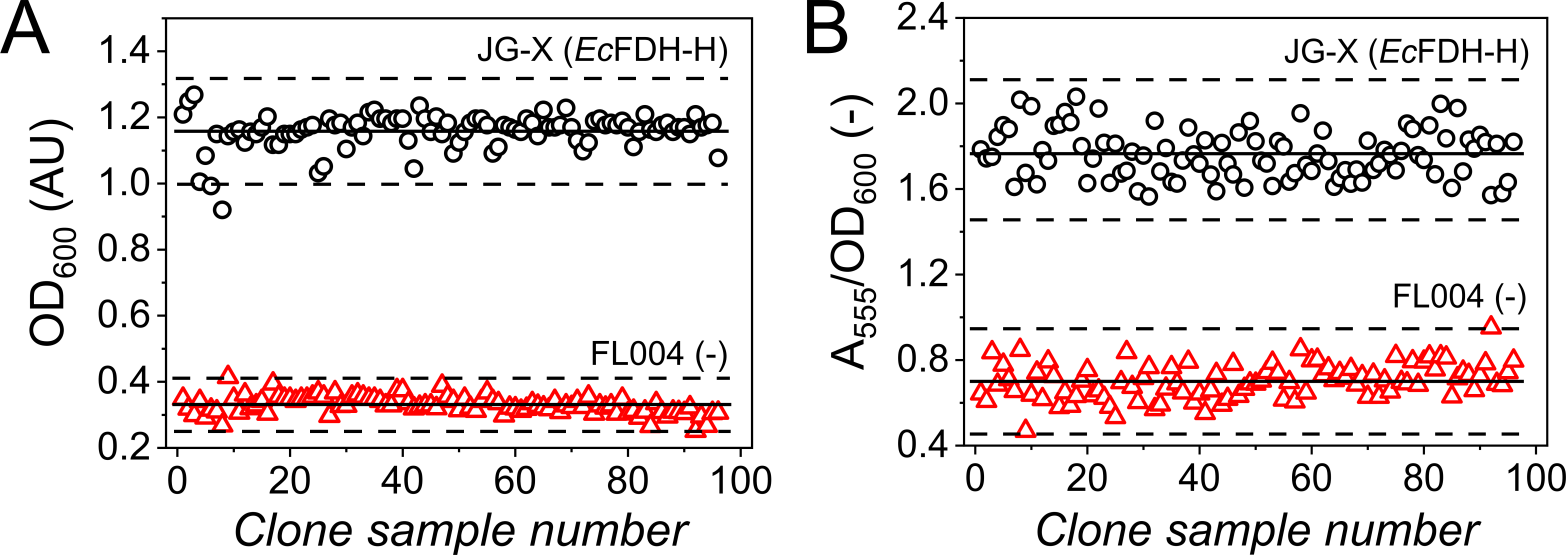
Signal distribution of sample clones during pre- and rescreening (A and B, respectively) of *E. coli* JG-X producing *Ec*FDH-H (WT) (black) and *Ec*FDH-H-deficient strain FL004 (red). Pre-screening of clones was performed by OD_600_ measurements in flat-bottom microtiter plates (MTPs) prior to which the bacterial cells were cultivated for 24 h at 30°C under an anaerobic atmosphere in a 250-µL glucose-supplemented growth medium in wells of a 500-µL deep-well plate. Sample preparation for the rescreening step included the collection of the cells contained in the pre-screened cell suspensions by centrifugation and their lysis by the addition of B-PER reagent. The formate dehydrogenase activity in the cell lysate was determined at λ = 555 nm using flat-bottom MTPs containing 5 µL of the cell lysate as well as formate and BV^2+^ dissolved in 50-mM phosphate buffer (pH 7.5) at concentrations of 10 and 2 mM, respectively. The average value (µ) and the set threshold (χ_0_ = µ ± 3 · σ) are displayed as solid and dashed lines, respectively.

Next, a screening assay was designed to screen an enzyme variant library in a platform that is presented schematically in Fig. 3. It consists of an initial pre-screening step that exploited the dependency of fermentative growth of *E. coli* on the catalytic activity of *Ec*FDH-H. In the case of impaired *Ec*FDH-H activity, also, the *in vivo* activity of the FHL is decreased resulting in a lower optical density during glucose fermentation. Clones reaching OD_600_ levels that were close to that of the parental strain were subjected to a consecutive colorimetric rescreening by a previously described enzyme assay (46, 47). In this second screening step, *Ec*FDH-H enzyme variants are directly assayed *in vitro* and improvements in catalytic activity can be resolved. Importantly, both screening stages can be performed in a 96-well plate format. Data variability in a screen is an important assay characteristic and should be minimized in order to be able to detect differences between assayed enzyme variants reliably. For the optimization of the screening assay, these parameters were determined as the coefficient of variance (CV) and *Z*′-factor (48) from additional growth trials performed with the two reference strains JG-X (*fdhF*) and FL004 (Δ*fdhF*). The optimal glucose concentration for the measurement of anaerobic cell growth as OD_600_ was identified as 4 g/L (Fig. S2B; Table S2). In order to assess the variability of the re- and pre-screening methods, the performance of both clones in the screen was analyzed by 96 replicate measurements. Growth experiments performed with *E. coli* JG-X indicated a CV of 0.046 (Fig. 4A), which is low in comparison to the 2.5-fold increase of the reached cell density from the control strain level and allows the reliable identification of actively growing *E. coli* strains for subjection to rescreening. Here, we determined the final OD_600_ of 1.01 as the lower threshold (χ_0_) based on the average final OD_600_ (µ) and standard deviation (σ) of the wild type (WT) according to the equation χ_0_ = µ – 3 · σ. This limit allowed us to exclude clones with an average final OD_600_ <0.74 and a CV of 0.083 from the second screening step at a low likelihood of false positives (*P* = 1.24 · 10^−5^; determined by the probability density function as described in Materials and Methods). The relation between the data variability and the overall signal range of an assay may be quantified using the *Z*′-factor that should ideally lie in the range of 0.5–1.0 (48). The *Z*′-factor for the investigated pre-screening assay was 0.65 indicating an ideal separation of OD_600_ data of the positive and negative control groups, i.e., *E. coli* strains JG-X and FL004, respectively.

The colorimetric rescreening was performed using *E. coli* cell lysates that were transferred into microtiter plate (MTP) wells containing the FDH substrates formate and benzyl viologen (BV^2+^). Enzymatic activity was monitored as the production of BV^+^ at λ = 555 nm. Statistical analysis of the obtained data (Fig. 4B) determined the CV value to be 0.66 for which samples with an average final A_555_/OD_600_ < 2.9 and a CV of 0.117 can be deselected at a low likelihood of false positives (*P* = 2.75 · 10^−5^; determined by the probability density function). To further complete the statistical analysis of the rescreening assay, the *Z*′-factor was determined as 0.51 validating the overall screening method. Overall, the throughput capacity of this assay is limited by the requirement of anaerobic conditions and was enhanced by the demonstrated screen robustness and effectiveness of the pre-screening stage for reducing the clone number subjected to rescreening. Based on the presented results, we estimate the upper limit of assay throughput to be 10^4^ *E. coli* clones per round without automation.

#### Semi-rational engineering of Ec*FDH-H* for increased catalytic activity

**Fig 5:**
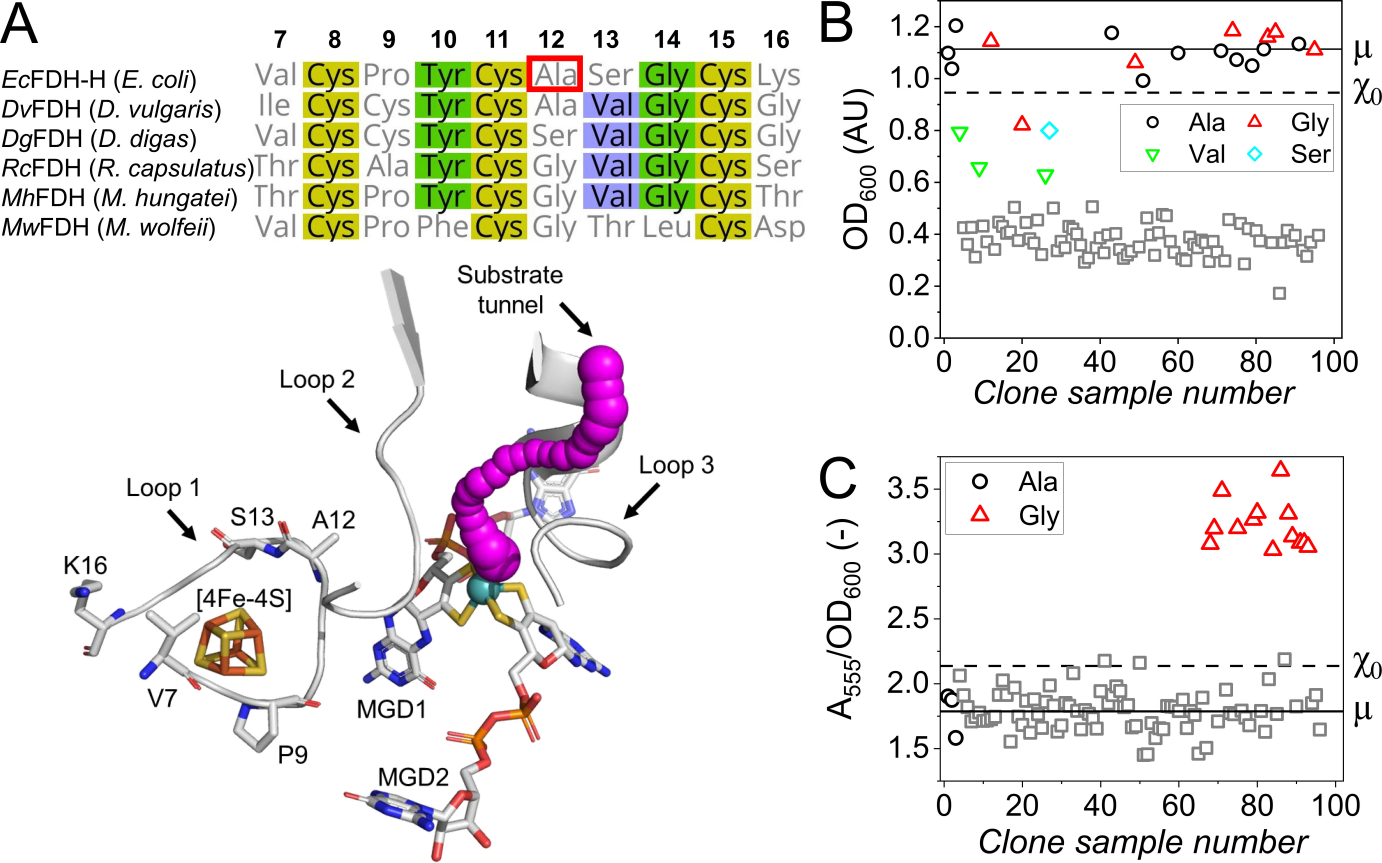
(**A**) Multi-sequence alignment of *Ec*FDH-H and bacterial metal-dependent FDHs [PDB IDs: 1FDO (*Ec*FDH-H), 6SDR (*Dv*FDH), 1H0H (*Dg*FDH), 6TG9 (*Rc*FDH), 7BKB (*Mh*FDH), and 5T5I (*Mw*FDH)] with decreasing degrees of homology indicated by yellow, green, and blue and residue positions in *Ec*FDH-H stated above the aligned sequences. The structure of the *Ec*FDH-H active-site region is displayed with side chains of substituted residues as well as the [4Fe-4S] cluster and molybdopterin guanine dinucleotide (MGD) cofactors in stick representation. The substrate tunnel providing substrate access to the *Ec*FDH-H active site (magenta filled-sphere array) was calculated and visualized using the CAVER PyMOL plugin (50) and the PyMOL Molecular Graphics System, version 1.7.2 Schrödinger, LLC. The results of pre- and rescreening of a variant library obtained by saturation mutagenesis of the codon encoding *Ec*FDH-H residue A12 (marked with a red box) are shown in panels (B and C), respectively. The *E. coli* strains JG-X and *Ec*FDH-H, respectively, are included for reference as clones 1–3. Only *E. coli* clones exceeding the pre-screening threshold (χ_0_, dashed line) were subjected to rescreening. The WT average (µ) is indicated as a solid line. The χ_0_ of the re- and pre-screening assay were calculated as χ_0_ = µ ± 3 · σ. Unidentified clones performing below χ_0_ are shown as gray squares.

For the purpose of testing the screening system, we prepared a library that probed the role of five amino acids in loop 1 because it is positioned between the Mo-cofactor of the *Ec*FDH-H active site and the [4Fe-4S] cluster a region (Fig. 5A) and may, therefore, provide interactions to neighboring enzyme regions involved in substrate binding and electron transfer. In order to minimize the risk of enzyme inactivation, residues with low conservation degrees were selected for substitution. These substitution targets were P9, A12, and S13 in the vicinity of the [4Fe-4S] cluster as well as V7 and K16 below the enzyme surface (Fig. 5A) and were investigated using a library comprised of 1,032 *Ec*FDH-H variants where each of the five positions was mutated individually. The size of this library was considered sufficiently large to reach an acceptable genetic variability based on a study by Reetz et al. who determined that a 95% coverage can be obtained with NNK codon degeneracy if a minimum of 94 clones are included for a single position (49). The growth-based pre-screening significantly reduced the library size from 1,032 to 96 clones and revealed that, among the tested positions, only the glycine substitution variant of the A12X variant library reached the set lower OD_600_ threshold of 0.95 (Fig. 5B). These clones clustered around an OD_600_ of 1.1 while most of the remaining clones exhibited retarded growth characterized by a low final OD_600_ of ~0.39. Notably, a few clones were scattered between these two extremes and identified by gene sequencing as variants A12V and A12S as well as a single false-negative clone producing *Ec*FDH-H variant A12G. The apparent partial retention of *Ec*FDH-H function in the case of A12V and A12S may be explained by the chemical similarity between the substituting side chains and the methyl group at the Ala α-carbon. This notion is supported by the fact that both, valine and serine, are common in homologous metal-dependent formate dehydrogenases or nitrate reductases at positions corresponding to residue A12 in *Ec*FDH-H (Fig. 5A). Notably, six out of the seven identified A12G clones reached OD_600_ values >χ_0_ indicating a high recognition probability for clones producing active *Ec*FDH-H variants.

The ability of selected clones to oxidize formate was assessed by the formate oxidation assay and identified the *Ec*FDH-H variant A12G as the only enzyme with an improved activity exceeding the *Ec*FDH-H (WT) conversion rate by a factor of 0.81 ± 0.10 (Fig. 5C). The purified *Ec*FDH-H-A12G oxidized formate at 534 ± 10 s^−1^ at saturating formate concentration and 2 mM BV^2+^, corresponding to an 82% increase compared to the wild-type enzyme (Fig. S4A), and bound formate more tightly than the native enzyme, which was apparent as change of $K_{m}^{Formate}$ from 2.36 ± 0.20 to 1.28 ± 0.08 mM. The binding affinity of the A12G variant for the soluble electron acceptor BV^2+^, on the other hand, was indistinguishable from the wild-type enzyme (Fig. S4B). Comparative molecular docking analysis suggested an alternative binding of formate in the active center via additional hydrogen bonds originating from residues H141 and R333 as a possible cause for the observed increased substrate binding affinity of the A12G variant (Fig. S5). Despite the A12G variant being more active than *Ec*FDH-H (WT) in the colorimetric rescreening step, their host *E. coli* cells reached a similar final cell density in the growth-based pre-screening step. The reason for this different trend could be that the supplied glucose was limiting cell growth during the pre-screening and thereby restricted the final observable cell density. Nevertheless, screening at an increased glucose titer is unlikely to increase the quality of the pre-screening assay because the final OD_600_ did not significantly change at glucose concentrations ≥ 4g/L (Fig. 2A). Another possible cause for the apparent upper detection limit of the pre-screening assay is the catalytic coupling between *Ec*FDH-H and HycE within the FHL complex. The conversion rate of its least active component limits catalysis of the overall reaction HCOO^−^ + H^+^ → CO_2_ + H_2_. This notion is supported by comparable gas production by strains JG-X [*Ec*FDH-H (WT)] and FL005 (*Ec*FDH-H-A12G) during anaerobic cultivation, being 761 ± 8 and 758 ± 32 mL, respectively. As there were no significant differences between the final cell densities reached by clones producing the catalytically enhanced A12G variant and the wild-type enzyme, a consecutive activity-based rescreening step is required to reliably identify the enzyme variants with improved activity.

### Conclusions

In summary, we present a robust growth-based screening strategy for the semi-rational engineering of *Ec*FDH-H that was validated through the identification of variant A12G exhibiting an 82% increased formate oxidation activity. The presented screening method relies on the growth-inhibiting effect of glucose fermentation products that rapidly accumulate in *Ec*FDH-H-deficient cells and are removed by the FHL. Therefore, the growth benefit provided by the FHL during fermentative growth may be employed to identify FHL components that completely or partially restore the activity of either of the two catalytic FHL centers and/or the iron**–**sulfur-cluster-mediated electron transfer between these active sites. In line with this mechanism, we envision the adaptation of this screening system to different FHL components and other electron-transferring enzyme complexes affecting microbial host fitness (51).

## MATERIALS AND METHODS

### Plasmids and strains

*E. coli* strain JG-X (MC4100, ∆*fdhF*, ∆*iscR*) harboring plasmids pTrc99a-*fdhF* (Amp^R^) and pSU21-*selC* (Chl^R^) was kindly provided to us by Prof. Shelley Minteer (Utah State University, Utah) (31). The strain JG-X was originally prepared by members of the Golbeck group (Pennsylvania State University, Pennsylvania) who have tested the MC4100 strain as well as the *iscR* and *fdhF* deletions and *selC* overexpression for the high-level production of *Ec*FDH-H as described in their Air Force Research Laboratory project report AFRL-OSR-VA-TR-2014-0088 (52). The *E. coli* strain MC4100 is a K-12 derivative with the genotype [*araD139*]*_B/r_* Δ[*argF-lac*]*169 λ^−^ e14- flhD5301 Δ(fruK-yeiR)725*(*fruA25*) *relA1 rpsL150*(strR) *rbsR22 Δ(fimB-fimE)632*(*::IS1*) *deoC1,* and its genome sequence has been published by Peters et al. (53). The *selC* gene of vector pSU-*selC* encodes the tRNA from which selenocysteyl-tRNA^Sec^ (54, 55) is synthesized and overexpressed to support the high-level production of Sec-containing *Ec*FDH-H. The vectors pFL004–pFL007 were used for *Ec*FDH-H variant production (D179E, D179L, K44R, and K44A, respectively) and were prepared by site-directed mutagenesis of the *fdhF* gene in vector pTrc99a-*fdhF* using a previously described method (46, 56) and complementary DNA primer pairs (Table S3). For *Ec*FDH-H variant production, pTrc99a-*fdhF* was eliminated from *E. coli* strain JG-X by repeated strain transfer (12 times) onto selective lysogeny broth (LB) agar (50 µg/mL chloramphenicol) yielding the strain FL003. The plasmid removal and absence of growth-inhibiting mutations were confirmed by replica-plating onto a double-selective solid medium containing ampicillin at 50 µg/mL as an additional antibiotic and comparative growth trials with the original and reconstituted parental strain JG-X (Fig. S6), respectively. The *Ec*FDH-H-deficient control strain FL004 and *Ec*FDH-H variant production strains FL005–FL009 were prepared by the transformation of electrocompetent cells of strain FL003 (*V* = 90 µL) with 1 µL of plasmid solution (*c* ≈ 50 ng/µL) of either insert-free pTrc99a (Nova Lifetech Ltd., Hong Kong) or pTrc99a-*fdhF* derivatives (Table 1), respectively. Electroporation of *E. coli* was performed using a MicroPulser electroporator (Bio-Rad, California) according to the manufacturer’s protocol and followed by a recovery step for which the cell suspension was supplemented with 800-µL SOC medium and incubated at 37°C for 1 h.

### *fdhf* mutant library construction

Site saturation mutagenesis of the *fdhF* gene was performed according to the previously described QuikChange method (57). In brief, the codon encoding the amino acid of interest on each strand was mutated in two parallel PCR reactions during a few cycles (typically five) using only the forward or reverse primer of a primer set specific for the chosen DNA locus. In the second stage, the two PCR reactions are combined, and the entire plasmid was amplified in the presence of the complete complementary primer pair during 20–30 additional thermocycles. The codons encoding *Ec*FDH-H residues V7, P9, A12, S13, and K16 were substituted with nucleotides encoding one of the other 19 common proteogenic amino acids. The used PCR reaction mix (*V* = 25 µL, Phusion PCR buffer) contained each dNTP at 0.1 mM, 200 nM degenerate primers (Table S3), 20 ng pTrc99a-*fdhF* template, and 0.02 U Phusion Hot Start II DNA polymerase (ThermoFisher Scientific, Massachusetts) and was subjected to five thermocycles including a 5-min primer extension step at 72°C. Subsequently, both reactions were combined, 0.02 U DNA polymerase was added, 20 thermocycles were performed, and the vector template was removed by incubation at 37°C for 2–3 h with 10 U added *Dpn*I restriction enzyme. TOP 10 electrocompetent cells were transformed with the pTrc99a-*fdhF* derivatives by combining a 100-µL cell suspension with 1 µL of the PCR product followed by electroporation, as described above, followed by the addition of 900-µL SOC medium and incubation at 37°C for 2 h for cell recovery. A 50-µL cell culture aliquot was spread on a selective LB agar medium and cultivated overnight at 37°C after which typically >400 colonies had formed. The cells were resuspended in 1-mL sterile ddH_2_O, and contained plasmids were isolated using the NucleoSpin plasmid isolation kit (Macherey-Nagel, Germany). The purified plasmids were used to transform *E. coli* FL003 cells (Table 1) by electroporation in a reaction mix consisting of 1-µL plasmid solution (~50 ng/µL) and 90-µL cell suspension, and cell recovery was performed by the addition of a 800-µL SOC medium followed by cultivation at 37°C for 1 h. Then, the cell suspension was diluted with ddH_2_O by a factor of 30–50, spread onto a solid selective LB medium, and incubated overnight at 37°C. In order to optimally represent the mutational diversity of the generated pTrc99a-*fdhF* derivatives in the clone library, colonies derived from a single mutation site were distributed into individual 2.0-mL wells of two 96-well deep-well MTPs by resuspension in 400-µL selective LB. Next, the wells were sealed with gas-permeable film and incubated at 37°C, 70% humidity, and 600 rpm for 24 h. These master plates were stored at −80°C following the addition of sterile glycerol solution to a final concentration of 23% (vol/vol). The coverage of the mutational diversity was assessed by sequencing of plasmids (Microsynth, Germany) that were purified from clones contained in nine randomly selected wells of each master plate using the E-Z 96 FastFilter Plasmid DNA kit (Omega Bio-Tek, Georgia).

### Bacterial cultivation in microtiter format for monitoring bacterial growth

Glycerol stocks of either *E. coli* JG-X or FL004 were back-diluted 1:50 in a 250-µL selective LB medium and incubated in 500-µL deep-well plates with conical bottoms (Eppendorf, Germany) for 24 h at 37°C and 600 rpm orbital shaking. The pre-culture was back-diluted 1:50 with a selective LB medium supplemented with 1 mM Na_2_MoO_4_ and 10 µM Na_2_SeO_3_, and *Ec*FDH-H production was induced by the addition of isopropyl-β-D-1-thiogalactopyranoside (IPTG) (Sigma Aldrich, Germany) to 100 µM according to a published protocol (31). Then, the deep-well plates were sealed with Easyseal transparent film (676001; Greiner Bio-one, Germany) and placed inside a Ziploc bag along with anaerobic indicator strips (ThermoFisher Scientific, Massachusetts) for transfer into a custom-made container (Fig. S7) containing palladium catalysts (Advanced Instruments, Massachusetts). The container was hermetically sealed, filled with anaerobic gas mix (95% N_2_, 5% H_2_), and incubated for 24 h at 30°C and 200 rpm. Microbial growth was monitored in an anaerobic cabinet as optical density at 600 nm in flat-bottom MTPs (ThermoFisher Scientific, Massachusetts) containing 200-µL culture samples using a Multiskan SkyHigh microplate reader (ThermoFisher Scientific, Massachusetts). The OD_600_ in MTP wells with an unknown pathlength was determined based on the absorbance measured at λ = 600 nm using the equation OD_600 nm_ = *A*_600 nm_/(1 cm · α). Here, α is the ratio between 1 cm and the length of the path that light travels through the 200-µL liquid sample in a MTP well and was determined empirically to be 3.57.

### Bacterial cultivation in 10-mL serum bottles for monitoring of growth medium acidification

Pre-cultivation of *E. coli* from glycerol stocks was carried out as described above. Cultivation trials were performed by first sealing sterile 10-mL serum bottles with butyl rubber stoppers in an anaerobic cabinet. The pre-culture was then back-diluted at a ratio of 1:50 in a 5-mL selective LB medium supplemented with 1 mM Na_2_MoO_4_, 10 µM Na_2_SeO_3_, 4 g/L glucose, and 100 µM IPTG and incubated for 18 h at 30°C and 200 rpm. During cultivation, serum bottles were removed from the incubator in 2-h intervals and sampled for pH determination using an AE150 Benchtop pH Meter (Fisher Scientific, Finland) and for spectroscopic analysis (OD_600_) in flat-bottom MTPs (200-µL sample volume) using a Multiskan SkyHigh microplate reader. Serum bottles sampled at timepoints 20, 22, and 24 h were inoculated 14 h past inoculation of the remaining serum bottles (sampled at 2–18 h).

### Bacterial cultivation in 1-L laboratory bottles for headspace analysis

Bacterial cultures were inoculated in an anaerobic cabinet by first transferring a 500-mL selective LB medium (50 µg/mL ampicillin, 50 µg/mL chloramphenicol) supplemented with 1 mM Na_2_MoO_4_, 10 µM Na_2_SeO_3_, 4 g/L glucose, and 100 µM IPTG into 1-L laboratory bottles (GL45; Schott, Germany) and adding 2.5 mL saturated *E. coli* pre-culture (preparation described above) followed by sealing with butyl rubber stoppers. The headspace of cultivation bottles was flushed with a stream of nitrogen gas for 10 min to remove hydrogen gas originating from the anaerobic cabinet. Cells were cultivated for 24 h at 30°C and 150 rpm followed by the discharging of headspace gas into a 1-L FlexFoil PLUS sample bag (SKC, UK). Gas chromatography was employed for the determination of the gas composition using an Agilent 490 Micro gas chromatograph (Agilent, California) equipped with two 10-m-long porous-layer open-tubular columns (0.32 mm inner diam.) coated either with a 30-µm-thick film of 5 Å molecular sieves (CP7535, Agilent, California) or a 10-µm-thick polar porous polymer (CP7580, Agilent, California). Gas sample components were separated in a stream of helium carrier gas and quantified with a micromachined thermal conductivity detector (Agilent, California) following calibration with standard gas mixtures. The total gas sample volume was determined by batch-wise removal of the sample bag contents with a graduated syringe.

### Screening system evaluation

The *Z*′-factor and CV of the MTP-based screening system were used to assess its quality and calculated as described previously using equations 1 and 2, respectively (48). Here, the mean μ and the standard deviation σ were determined from the cell densities reached by the positive and negative control strains being *E. coli* JG-X and FL004, respectively:


(1) $$Z\prime \;factor=1-\left[\frac{3\cdot \left(\sigma_{pos}+\sigma_{neg}\right)}{\mu_{pos}-\mu_{neg}}\right]$$

(2) $$CV=\frac{\sigma }{\mu }$$

### Growth-based pre- and colorimetric rescreening

Growth-based pre-screening was performed using the above-mentioned method for bacterial cultivation in microtiter format. Clones displaying higher final OD_600_ than the set threshold of the parental strain *E. coli* JG-X: χ_0_ = µ – 3 · σ were selected as active variants and analyzed further by the colorimetric rescreening as follows. Cells were cultured in 2.5-mL deep-well plates anaerobically as described above and sealed with Easyseal transparent film (676001; Greiner Bio-one, Germany) inside an anaerobic cabinet. Bacterial cells were harvested by centrifugation for 10 min at 4°C and 4,000 × *g*. The plates were then transferred into an anaerobic chamber, where the supernatant was removed and the cell pellet was resuspended in the 50-µL degassed B-PER cell lysis reagent containing 100 µg/mL of lysozyme (ThermoFisher Scientific, Massachusetts). The lysis mix was agitated for 30 min at room temperature and sampled for colorimetric rescreening. Here, a 5-µL cell lysate sample was transferred into a flat-bottom MTP well along with 185 µL of *Ec*FDH-H reaction mix containing the *Ec*FDH-H substrates formate and BV^2+^ dissolved in 50-mM potassium phosphate buffer (pH 7.5) at a concentration of 10 and 2 mM, respectively. The chromogenic reaction mixture was incubated for 5 min at 25°C after which its absorbance was recorded at 555 nm (*A*_555_) using a Multiskan SkyHigh microplate reader (ThermoFisher Scientific, Massachusetts). Clones exhibiting higher specific formate oxidation activity than the set activity threshold of *E. coli* JG-X (χ_0_ = µ + 3 · σ), as apparent from the *A*_555_/OD_600_ ratio, were selected for *fdhF* gene sequencing to identify the encoded *Ec*FDH-H variant.

### Statistical analysis

To assess the quality of the pre- and rescreening methods, the probability of selecting false-positive clones was determined at a standard distribution of the measured parameter (final OD_600_ reached during fermentative growth or cell-mass specific FDH enzyme activity) at a given hypothetical average value. Here, false positives are colonies belonging to a clone that perform above the set threshold in the used assay and whose average of all colonies belonging to the same clone is below the threshold. This analysis was based on a hypothetical clone library with a given average value of the monitored parameter (µ) around which the data of all samples of the same clone library are distributed according to a standard distribution. The CV value of this sample was assumed to be identical to the one determined for the strain FL004 analyzed by either the pre- or re-screening method. The probability of identifying false-positive clones at a given clone-characteristic average value was obtained using the probability density function (equation 3):


(3) $$f\left(x\right)=\frac{1}{\sqrt{2\pi \sigma }}e^{-\frac{1}{2}{\left(\frac{\chi 0-\mu }{\sigma }\right)}^{2}}$$

### Alignment of *Ec*FDH-H homologs

Amino acid sequences of proteins with >30% sequence homology with *Ec*FDH-H (PDB ID: 1FDO) were identified in the RCSB Protein Data Bank and aligned to *Ec*FDH-H using the Clustal Omega tool (58) included in the bioinformatics software Geneious Prime (Biomatters, New Zealand).

## Data Availability

Data used for the figures and supplemental tables are available upon request.

## AFTER EPUB

*[This article was published on 28 August 2024 with an error in equation 2. The equation was corrected in the current version, posted on 30 August 2024.]*

## SUPPLEMENTAL MATERIAL

Supplemental material aem.01472-24-s0001.pdf Tables S1 to S3; Figures S1 to S7.

ASM does not own the copyrights to Supplemental Material that may be linked to, or accessed through, an article. The authors have granted ASM a non-exclusive, world-wide license to publish the Supplemental Material files. Please contact the corresponding author directly for reuse.
