# Supplementary material for: A growth-based screening strategy for engineering the catalytic activity of an oxygen-sensitive formate dehydrogenase
Source: Appl Environ Microbiol. 2024 Aug 28;90(9):e01472-24. doi: 10.1128/aem.01472-24 (PMC11409667; doi:10.1128/aem.01472-24)
Supplement: Supplemental material — Tables S1 to S3; Figures S1 to S7. [file aem.01472-24-s0001.pdf]

Supporting information

A growth-based screening strategy for engineering the catalytic activity of an oxygen-sensitive formate dehydrogenase

Feilong Li[1, 2], Silvan Scheller[1] and Michael Lienemann[2, *]

[1] Department of Bioproducts and Biosystems, Aalto University, 02150 Espoo, Finland

[2] VTT Technical Research Centre of Finland Ltd., 02150 Espoo, Finland

*Corresponding author: Michael.lienemann@vtt.fi

Supplementary tables

Table S1. Gas composition of *Escherichia coli* culture headspace after 24 h anaerobic cultivation in LB medium containing 4 g/L glucose. The gas component contents were determined from the area of each gas chromatogram signal peak. The displayed means and standard deviations were determined from biological replicates (n = 3).

Gas component*	Content in gas headspace of cell cultures of *E. coli* strains [%]	
	JG-X	FL004
CO_2 (0.481 min)	25.8 ± 0.4	4.98 ± 0.08
H_2 (0.580 min)	22.1 ± 0.5	0.22 ± 0.02

*GC-Retention time indicated in brackets

Table S2. *Z′* factor determined from distribution of cell densities, as apparent from the optical density at λ = 600 nm, reached after anaerobic cultivation of *E. coli* strains JG-X and FL004 for 24 h in LB medium at different glucose concentrations. Each data point represents three biological replicates.

$c_{Glucose}$ (g/L)	*Z′* factor
20	0.68
10	0.68
4.0	0.67
2.0	0.50
1.0	0.40
0.50	0.36
0.20	0.41
0.10	0.27
0.050	0.28
0.020	0.21
0.010	0.37
0.0	0.07

Table S3. Nucleotide sequences of primers used for site-saturation mutagenesis (SSM) and site-directed mutagenesis (SDM) of the *E. coli fdhF* gene. The sequences were designed according to Liu et al. (1) using the Primer X online tool (https://www.bioinformatics.org/primerx/index.htm). Nucleotides are grouped as *Ec*FDH-H encoding-codons with mutated nucleotides shown in red (K = guanine or thymine, M = adenine or cytosine, N = adenine or guanine or cytosine or thymine).

Primer name	Nucleotide sequence (5' to 3' terminus)
V7X_SSM_F	G AAA AAA GTC GTC ACG NNK TGC CCC TAT TGC GCA TC
V7X_SSM_R	GA TGC GCA ATA GGG GCA MNN CGT GAC GAC TTT TTT C
P9X_SSM_F	GTC GTC ACG GTT TGC NNK TAT TGC GCA TCA GG
P9X_SSM_R	CC TGA TGC GCA ATA MNN GCA AAC CGT GAC GAC
A12X_SSM_F	CG GTT TGC CCC TAT TGC NNK TCA GGT TGC AAA ATC AAC
A12X_SSM_R	GTT GAT TTT GCA ACC TGA MNN GCA ATA GGG GCA AAC CG
S13X_SSM_F	GTT TGC CCC TAT TGC GCA NNK GGT TGC AAA ATC AAC CTG
S13X_SSM_R	CAG GTT GAT TTT GCA ACC MNN TGC GCA ATA GGG GCA AAC
K16X_SSM_F	C TAT TGC GCA TCA GGT TGC NNK ATC AAC CTG GTC GTC GAT AA
K16X_SSM_R	TT ATC GAC GAC CAG GTT GAT MNN GCA ACC TGA TGC GCA ATA G
K44A_SDM_F	G GGT ACC CTG TGT CTG GCA GGT TAT TAT GGC TGG G
K44A_SDM_R	C CCA GCC ATA ATA ACC TGC CAG ACA CAG GGT ACC C
K44R_SDM_F	G GGT ACC CTG TGT CTG CGG GGT TAT TAT GGC TGG G
K44R_SDM_R	C CCA GCC ATA ATA ACC CCG CAG ACA CAG GGT ACC C
D179E_SDM_F	G TAC AAC CCG GCG GAG TCC CAC CCA ATC GTG
D179E_SDM_R	CAC GAT TGG GTG GGA CTC CGC CGG GTT GTA C
D179L_SDM_F	G TAC AAC CCG GCG CTG TCC CAC CCA ATC G
D179L_SDM_R	C GAT TGG GTG GGA CAG CGC CGG GTT GTA C

Supplementary figures

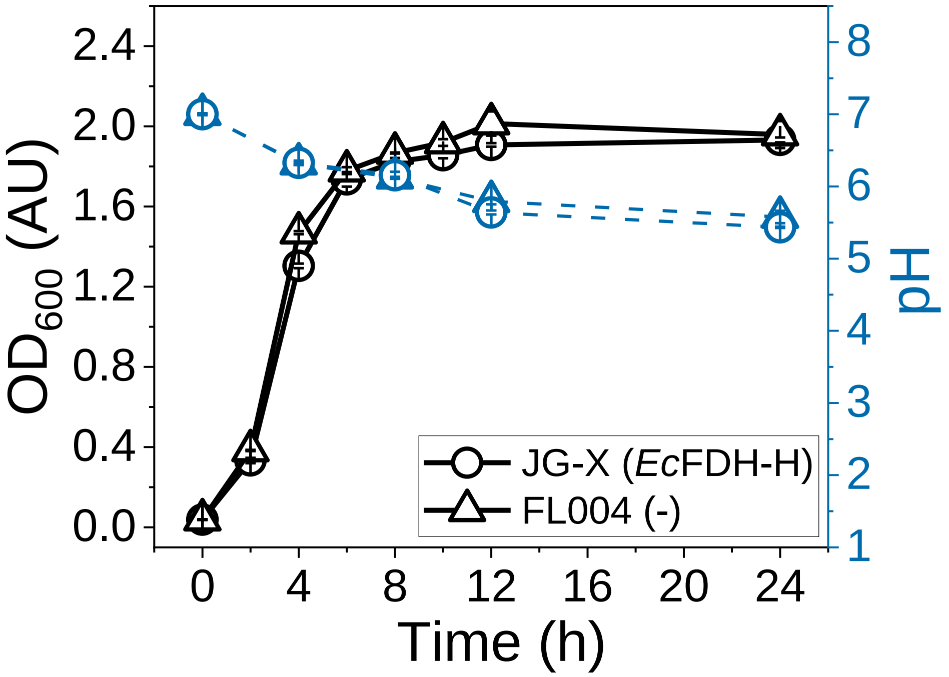

Figure S1. Aerobic growth and growth medium acidification of *E. coli* strains JG-X (*Ec*FDH-H) and FL004(-) in 10 mL glucose-supplemented LB medium (4 g/L) containing 1 mM Na_2MoO_4, 10 μM Na_2SeO_3, and 100 μM IPTG to support overproduction of *Ec*FDH-H. The cultivation was performed in 50-mL Erlenmeyer flasks at 30°C and 200 rpm orbital shaking.

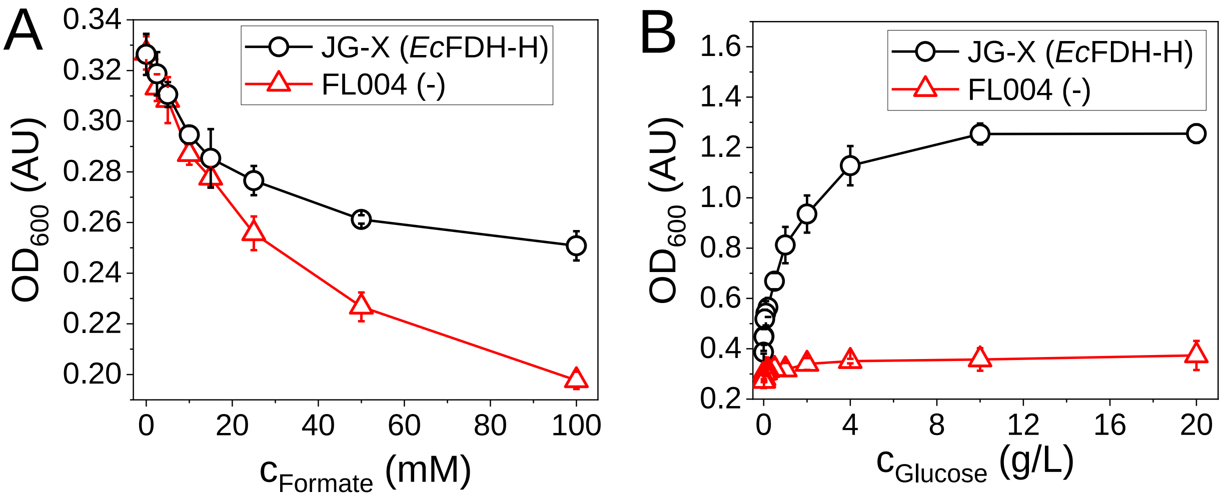

Figure S2. *E. coli* cell densities reached after 24 h incubation of *Ec*FDH-H-producing strain JG-X and *Ec*FDH-H-deficient strain FL004 under an anaerobic atmosphere in LB medium containing 1 mM Na_2MoO_4, 10 µM Na_2SeO_3, and 100 µM IPTG as well as different concentrations of (**A**) formate and (**B**) glucose in the presence of 15 mM formate. The cell cultivation was performed at 30°C and 600 rpm orbital shaking for 24 h in 96-well deep well plates inside an anaerobic container. The displayed mean values and standard deviations were determined from triplicate measurements.

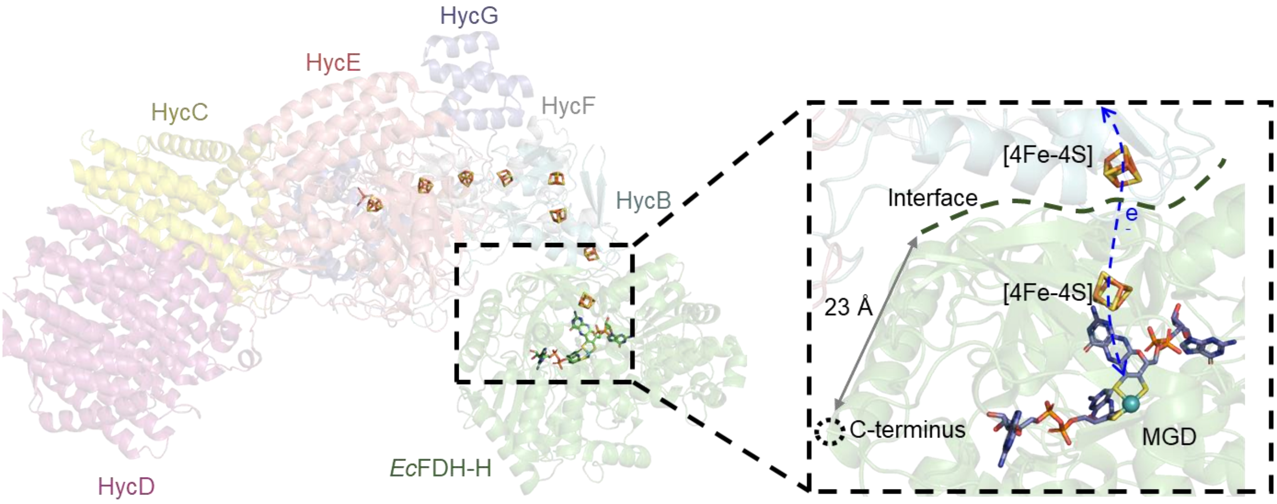

Figure S3. Structure of the *E. coli* formate hydrogenlyase complex (PDB ID: 7Z0T) and location of the *Ec*FDH-H C-terminus with respect to the *Ec*FDH-H–HycB interface. Bound electron-transferring iron-sulfur clusters, the *bis*-molybdopterin guanine dinucleotide (MGD) cofactor of the *Ec*FDH-H subunit and the [NiFe] cofactor of HycE are shown in stick representation. *Ec*FDH-H is denoted as FdhF in the original structural characterization of the enzymatic complex by Steinhilper et al.(2).

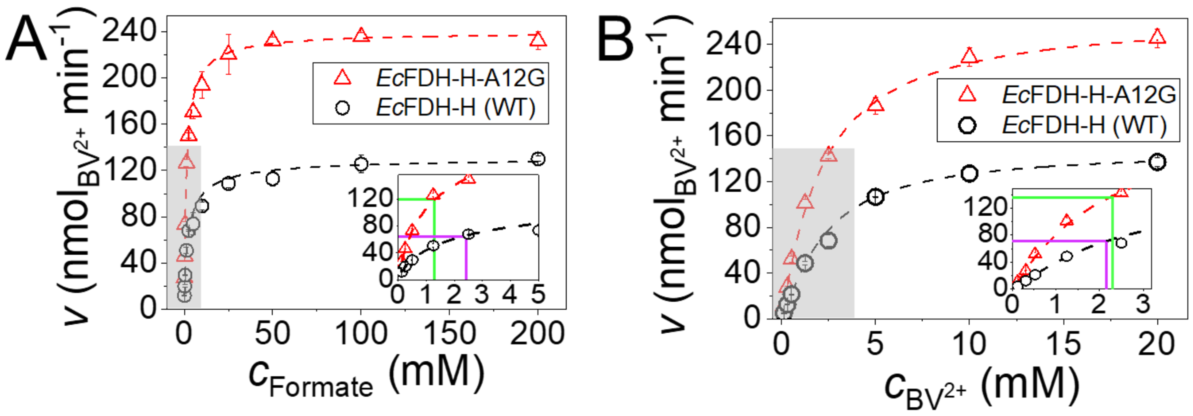

Figure S4. Rate of benzyl viologen dication (BV^{2+}) reduction by *Ec*FDH-H (WT) and variant A12G at variable concentrations of formate (**A**) and BV^{2+} (**B**) at 25°C under anaerobic conditions. The reaction mix had a volume of 200 µL and was prepared by combining 185 µL 50 mM potassium phosphate buffer (pH 7.5) with 5 µL enzyme solution (60 µg/mL), 5 µL of dissolved formate (2.0 mM – 8.0 M) and 5 µL BV^{2+} solution (0.40 – 800 mM). The final concentration of one of the co-substrates was kept constant at either 2 mM (BV^{2+}) or 10 mM (formate). The reaction was monitored as change of A_{555} and the apparent kinetic parameters $K_m^{Formate}$ and $K_m^{BV^{2+}}$ of both enzymes (x-axis intersections with coloured lines in insets) were determined by fitting of the kinetic data to the Michaelis-Menten equation (dashed lines). Error bars represent the standard deviation of three independent experiments.

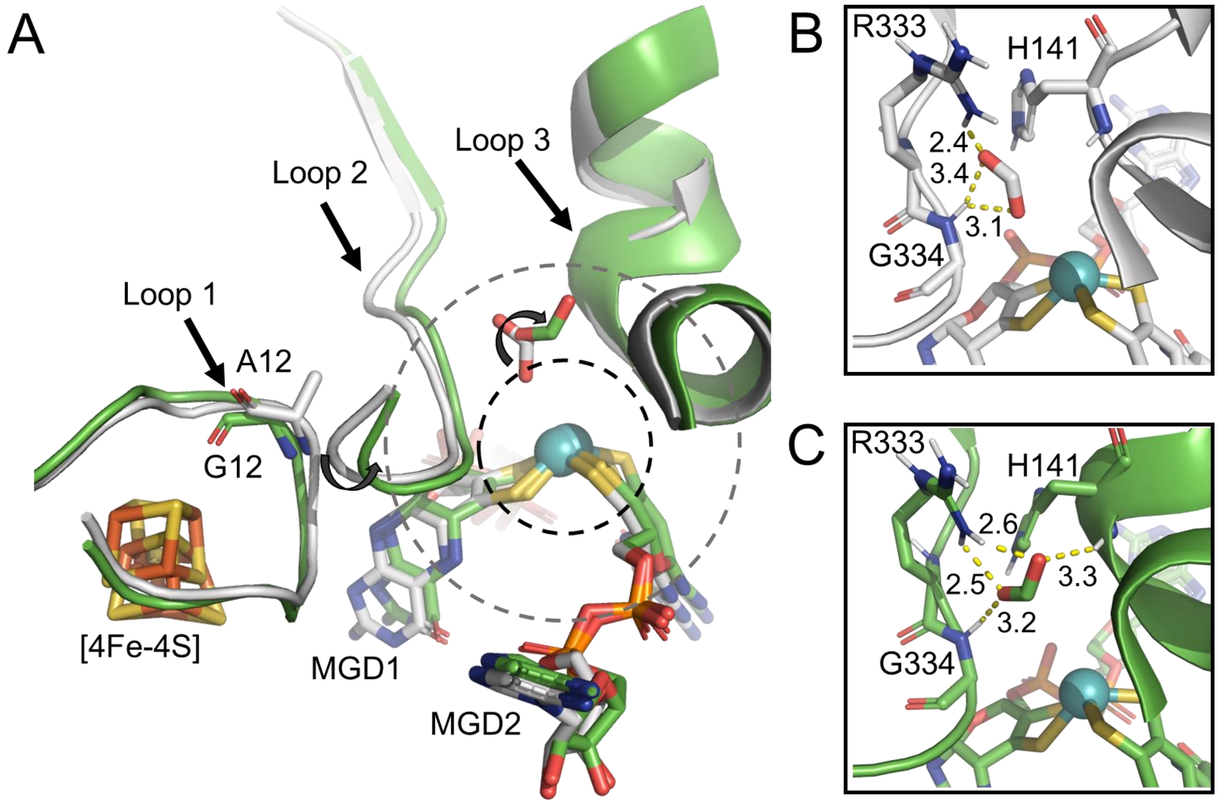

Figure S5. Formate binding in the active site of *Ec*FDH-H (WT) (grey) and -A12G variant (green) as predicted by molecular docking. The figure presents the superimposed *Ec*FDH-H (WT)–formate complex (**A**) and active-site structures of either the *Ec*FDH-H (WT)–formate complex (**B**) or the modelled *Ec*FDH-H-A12G–formate complex (**C**). MGD cofactors, [4Fe-4S] cluster, amino acid residues and formate molecule are shown in stick representation. The lengths of hydrogen bonds (yellow dashed lines) between residues R333, G334, and H141 and formate are stated in angstroms (Å). Dashed circles indicate inner and outer Mo-coordination spheres of the catalytic Mo-atom (cyan) according to Nazemi et al. (3). The structure of *Ec*FDH-H-A12G was modelled using the MODELLER tool (4) in Discovery Studio 2019 (BIOVIA, CA) with *Ec*FDH-H (PDB ID: 1FDO) as template. The model with the lowest discrete optimised protein energy score was selected and used for docking. The formate structure was retrieved from the PubChem database (https://pubchem.ncbi.nlm.nih.gov/), energy-minimized using the In Situ Ligand Minimization tool and docked into the *Ec*FDH-H structure using CDOCKER (Discovery Studio 2019) with a 16-Å-diameter docking sphere around the Mo ion using a top hit value of 1 and a pose cluster radius of 0.5. The number of random conformations was set to 100. The enzyme–formate complex structures were minimized by 960 steps of steepest descent with an RMS gradient tolerance of 3, followed by conjugate gradient minimization in 960 steps and displayed using the PyMOL Molecular Graphics System, Version 1.7.2 Schrödinger, LLC.

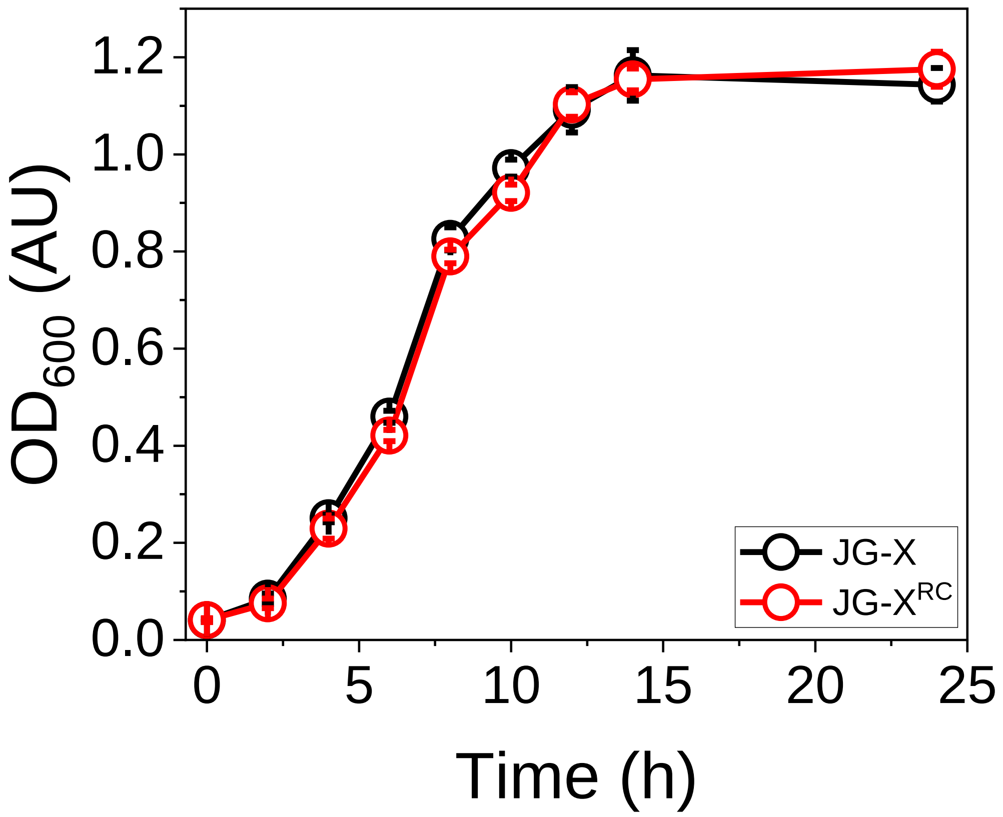

Figure S6. Growth profiles of parental and reconstituted *E. coli* strain JG-X (JG-X and JG-X^{RC}, respectively) recorded during anaerobic cultivation in 30-mL serum bottles containing 10 mL LB medium supplemented with 4 g/L glucose, 1 mM Na_2MoO_4, 10 µM Na_2SeO_3, and 100 µM IPTG for overproduction of recombinant *Ec*FDH-H. The cell cultivation was performed at 30 °C and 200 rpm orbital shaking for 24 h. Strain JG-X^{RC} was prepared by transformation of the *E. coli* strain FL003 with the plasmid pTrc99a-*fdhF*.

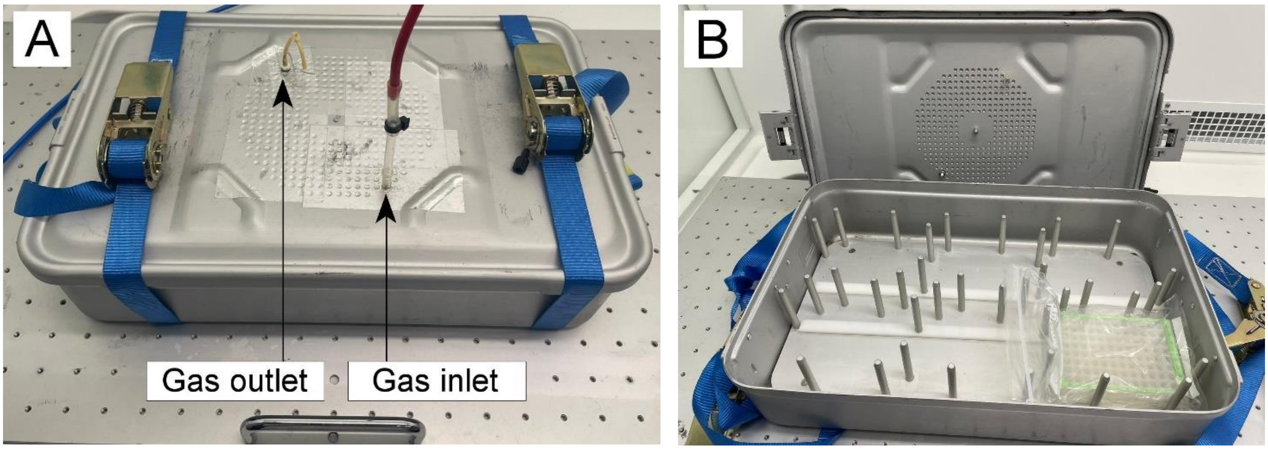

Figure S7. Sterilization aluminium container used for anaerobic cultivation of *E. coli* strains in microtiter-plate format. (**A**) Closed container mounted on shaker support plate. To ensure gas-tightness, ventilation openings in container lid are sealed with aluminium seal tape and silicon grease is applied to the sealing rubber ring of the lid. (**B**) Pin assembly inside container supporting the cultivation plate.

Supplementary references

1. Liu H, Naismith JH. 2008. An efficient one-step site-directed deletion, insertion, single and multiple-site plasmid mutagenesis protocol. BMC Biotechnol 8:91.
2. Steinhilper R, Hoff G, Heider J, Murphy BJ. 2022. Structure of the membrane-bound formate hydrogenlyase complex from Escherichia coli. Nat Commun 13:5395.
3. Nazemi A, Steeves AH, Kastner DW, Kulik HJ. 2022. Influence of the greater protein environment on the electrostatic potential in metalloenzyme active sites: The case of formate dehydrogenase. J Phys Chem B 126:4069-4079.
4. Sali A, Blundell TL. 1993. Comparative protein modelling by satisfaction of spatial restraints. J Mol Biol 234:779-815.
